# Poor prognostic and staging value of tumor deposit in locally advanced rectal cancer with neoadjuvant chemoradiotherapy

**DOI:** 10.1002/cam4.2034

**Published:** 2019-02-21

**Authors:** Yaqi Wang, Jing Zhang, Menglong Zhou, Lifeng Yang, Juefeng Wan, Lijun Shen, Liping Liang, Ye Yao, Hui Zhang, Zhen Zhang

**Affiliations:** ^1^ Department of Radiation Oncology Fudan University Shanghai Cancer Center Shanghai China; ^2^ Department of Oncology Shanghai Medical College Fudan University Shanghai China

**Keywords:** locally advanced rectal cancer, neoadjuvant chemoradiotherapy, prognosis, staging, tumor deposit

## Abstract

Tumor deposit (TD) was associated with poor survival in colorectal cancer. However, its prognostic and staging value in locally advanced rectal cancer (LARC) patients following neoadjuvant chemoradiotherapy (neo‐CRT) is controversial. Four hundred and ninety‐five LARC patients following neo‐CRT and surgery were retrospectively analyzed. Univariate and multivariate analyses were performed using Kaplan‐Meier method and Cox proportional hazards regression in all lymph node (LN) ‐negative and LN‐positive patients. Next, we used three methods to classify the counts of LNs and TDs (oN, only LN counts; n1N, counts according to the N1c standards; n2N, total counts of LNs and TDs) to evaluate the impact of TD on N staging. TD‐positive patients were associated with more aggressive clinicopathological features. In multivariate analyses, TD was an independent poor prognostic factor of overall survival (OS), disease‐free survival (DFS), and local recurrence‐free survival in all patients. In LN‐negative patients, TD was an independent poor prognostic factor of OS, DFS and distant metastasis‐free survival (DMFS). In LN‐positive patients, TD has poor prognostic value only in patients with one positive LN. Three multivariate analyses according to three N staging methods showed that oN was not an independent prognostic factor, whereas n1N and n2N were independently associated with poor survival in OS, DFS and DMFS. The n2N method seemed to be better than n1N method. TD is an independent poor prognostic factor in LARC patients following neo‐CRT, especially in patients with no more than one positive LN. TD probably should be considered as one positive LN when performing N staging.

## 1 INTRODUCTION

Colorectal cancer is the second leading cause of cancer deaths in the United States.^1^ Neoadjuvant chemoradiotherapy (neo‐CRT) followed by total mesorectal excision is the standard care for patients with locally advanced rectal cancer (LARC).^2^, ^3^, ^4^ The combination of radiation with capecitabine significantly improved local control and local recurrence‐free survival but did not improve metastasis‐free survival or overall survival.^5^ Tumor deposits (TDs) are found in the perirectal and mesenteric adipose tissue around rectal adenocarcinomas. Several editions of the American Joint Committee on Cancer (AJCC) staging manual have defined TDs. The current eighth edition classifies TD as follows: the deposit should be in the pericolorectal fat or adjacent mesocolic fat away from the leading edge of the tumor with no evidence of residual lymph node tissue and within the lymph drainage area of the primary carcinoma. Previously, several studies reported that TDs were associated with decreased prognosis and may identify patients with more aggressive tumors who require aggressive treatment.^6^ According to the eighth edition of AJCC stating guidelines, patients with positive TDs but negative lymph nodes (LNs) are classified as N1c. However, TD is not included in the N category for patients with positive LNs.

However, patients recruited in these studies did not receive neo‐CRT. Some changes will occur after CRT, such as decreased number of TDs, degenerative tumor cells, the formation of mucous lakes and tissue fibrosis.^7^ Thus, the prognostic and staging values of TDs in LARC patients who received neo‐CRT remain unclear. The present study aimed to evaluate the prognostic significance of TDs in LARC patients after neo‐CRT, verify the applicability of the N1c category in those tumors, and explore the appropriate methods of N staging for those patients.

## 2 PATIENTS AND METHODS

### 2.1 Study population

Between January 2006 and March 2015, a consecutive cohort of 550 patients with locally advanced (cT3‐4 and/or cN1‐2) rectal adenocarcinoma treated with neo‐CRT followed by surgery at the Fudan University Shanghai Cancer Center were retrospectively analyzed. Patients with any of the following criteria were excluded from the present study: (a) patients with multiple primary malignancies, (b) patients who died in the immediate postoperative period (within 30 days), (c) patients with distant metastasis identified preoperatively, (d) patients with incomplete pathological data, (e) patients with an interval from the completion of radiation to surgery greater than 16 weeks and (f) patients who were lost to follow‐up. After considering the above criteria, a total of 495 LARC patients were included in our study.

### 2.2 Data collection

Multiple clinicopathological data were obtained, including number of TDs, age, gender, depth of invasion (pre‐CRT and postoperation), number of metastatic LNs (pre‐CRT and postoperation), distance from the anus, radiation dose, interval chemotherapy (chemotherapy within the interval between the neoadjuvant chemoradiotherapy and surgery, usually used during 2009‐2015 in our center despite of those with older age, poor body condition or other reasons), operation procedure, adjuvant chemotherapy, differentiation grade, TRG score, number of LNs examined, and invasion status of nerves, vessels and circumferential resection margins (CRM). TDs were defined and evaluated based on the eighth edition of the AJCC staging manual. The four‐point TRG system is graded on a scale of 0 (complete response; no viable cancer cells) to 3 (poor response; minimal or no regression, extensive residual cancer). Survival data, including overall survival (OS), disease‐free survival (DFS), distant metastasis‐free survival (DMFS), and local recurrence‐free survival (LRFS), were recorded after the follow‐up.

### 2.3 N category methods

In this study, we used three methods of N categorization (oN, n1N, and n2N) to evaluate the impact of the TD count on tumor staging methods. The following three methods were employed (Table S1). (a) oN method, N staging by counting the number of metastatic LNs without TDs. It was the old method used before the seventh edition AJCC staging system. (b) n1N method, N staging according to the N1c category of the eighth edition AJCC staging system. It is the new method used currently in CRC patients without neo‐CRT. We would like to verify its applicability in LARC patients with neo‐CRT. (c) n2N method, N staging by counting the total number of metastatic LNs and TDs, considering one TD as one metastatic LN. It is the new method that we would like to compare with n1N method and explore the overall value of TD count on N staging.

### 2.4 Statistical analysis

Data for all categorical variables were summarized as frequencies, and data for all continuous variables were presented as medians and ranges. The chi‐square tests were used to compare differences in the distributions and proportions of the clinicopathological variables based on TD status. Survival curves were calculated using the Kaplan‐Meier method, and survival functions were compared using the log‐rank test. Univariate and multivariate Cox proportional hazards regression analyses were used to estimate the association between TD status and outcomes (OS, DFS, LRFS, DMFS) in all patients including, LN‐negative patients and LN‐positive patients. Clinicopathological factors with *P*‐values < 0.1 in univariate analyses were included in the multivariate analyses. Differences were considered statistically significant for *P*‐values < 0.05. All statistical analyses were performed using SPSS version 22.0 (SPSS, Inc., Chicago, IL).

## 3 RESULTS

### 3.1 Patient characteristics

A total of 495 LARC patients were included in the study. According to the criteria of eighth edition of the AJCC staging manual, 82.2% (407 of 495) of the patients were TD‐negative, and 17.8% (88 of 495) were TD‐positive. Patient characteristics based on TD groups are listed in Table 1. Compared with TD‐negative patients, TD‐positive patients were more likely to be younger (< 50 years old: 42.0% [37 of 88] vs 30.2% [123 of 407], *P *=* *0.032) and exhibit higher cN categories (cN2: 59.1% [52 of 88] vs 39.6% [161 of 407], *P *=* *0.002). Pathological results revealed that TD‐negative cases were more likely to exhibit lower degrees of TRG scores (TRG = 0: 24.8% [101 of 407] vs 4.5% [4 of 88], *P *<* *0.001) and ypT (ypT0: 24.8% [101 of 407] vs 4.5% [4 of 88], *P *<* *0.001) and ypN (ypN0: 75.4% [307 of 407] vs 45.5% [40 of 88], *P *<* *0.001) categories. In addition, compared with TD‐negative patients, TD‐positive patients exhibited an increased frequency of vascular invasion (11.4% [10 of 88] vs 5.4% [22 of 407], *P *=* *0.039) and neural invasion (20.5% [18 of 88] vs 8.6% [35 of 407], *P *=* *0.001). The above findings indicated that TD‐positive LARC patients tended to exhibit worse malignant clinicopathological features despite receiving neo‐CRT.

**Table 1:** Distribution of clinicopathological features for all LARC patients stratified by TD subgroups

Characteristics, No. (%)	All (*N* = 495)	TD‐negative (*N* = 407)	TD‐positive (*N* = 88)	*P*‐value
Age (y)				0.032
<50	160 (32.3)	123 (30.2)	37 (42.0)	
≥50	335 (67.7)	284 (69.8)	51 (58.0)	
Gender				0.353
Male	352 (71.1)	293 (72.0)	59 (67.0)	
Female	143 (28.9)	114 (28.0)	29 (33.0)	
cT				0.115
T2	11 (2.2)	11 (2.7)	0 (0.0)	
T3	387 (78.2)	312 (76.7)	75 (85.2)	
T4	97 (19.6)	84 (20.6)	13 (14.8)	
cN				0.002
N0	61 (12.3)	56 (13.8)	5 (5.7)	
N1	221 (44.6)	190 (46.7)	31 (35.2)	
N2	213 (43.0)	161 (39.6)	52 (59.1)	
Distance from anus (cm)				0.560
≤5	290 (58.6)	236 (58.0)	54 (61.4)	
>5	205 (41.4)	171 (42.0)	34 (38.6)	
Radiation dose (Gy)				0.445
≤50	396 (80.0)	323 (79.4)	73 (83)	
>50	99 (20.0)	84 (20.6)	15 (17.0)	
Interval chemotherapy				0.339
No	175 (35.4)	140 (34.4)	35 (39.8)	
Yes	320 (64.6)	267 (65.6)	53 (60.2)	
Surgical procedure				0.828
APR	267 (53.9)	222 (54.5)	45 (51.1)	
AR	194 (39.2)	157 (38.6)	37 (42.0)	
Hartmann	34 (6.9)	28 (6.9)	6 (6.8)	
Adjuvant chemotherapy				0.575
No	35 (7.1)	30 (7.4)	5 (5.7)	
Yes	460 (92.9)	377 (92.6)	83 (94.3)	
Differentiation grade				0.024
Low	65 (13.1)	45 (11.1)	20 (22.8)	
Middle	203 (41.0)	167 (41.1)	36 (40.9)	
High	16 (3.2)	13 (3.2)	3 (3.3)	
Unknown	210 (42.5)	181 (44.6)	29 (33.0)	
TRG				<0.001
0	105 (21.2)	101 (24.8)	4 (4.5)	
1	133 (26.9)	109 (26.8)	24 (27.3)	
2	222 (44.8)	172 (42.3)	50 (56.8)	
3	35 (7.1)	25 (6.1)	10 (11.4)	
ypT				<0.001
T0	105 (21.2)	101 (24.8)	4 (4.5)	
T1	20 (4.0)	20 (4.9)	0 (0.0)	
T2	138 (27.9)	117 (28.7)	21 (23.9)	
T3	203 (41.0)	147 (36.1)	56 (63.6)	
T4	29 (5.9)	22 (5.4)	7 (8.0)	
ypN				<0.001
N0	347 (70.1)	307 (75.4)	40 (45.5)	
N1	100 (20.2)	74 (18.2)	26 (29.5)	
N2	48 (9.7)	26 (6.4)	22 (25.0)	
LNs examined				0.155
≤11	287 (58.0)	230 (56.5)	57 (64.8)	
>11	208 (42.0)	177 (43.5)	31 (35.2)	
Vascular invasion				0.039
Negative	463 (93.5)	385 (94.6)	78 (88.6)	
Positive	32 (6.5)	22 (5.4)	10 (11.4)	
Neural invasion				0.001
Negative	442 (89.3)	372 (91.4)	70 (79.5)	
Positive	53 (10.7)	35 (8.6)	18 (20.5)	
CRM invasion				0.419
Negative	492 (99.4)	404 (99.3)	88 (100.0)	
Positive	3 (0.6)	3 (0.7)	0 (0.0)	

TD, tumor deposit; LN, lymph node; TRG, tumor regression grade; CRM, circumferential resection margin.

### 3.2 Prognostic value of tumor deposits in all patients

**Figure 1:**
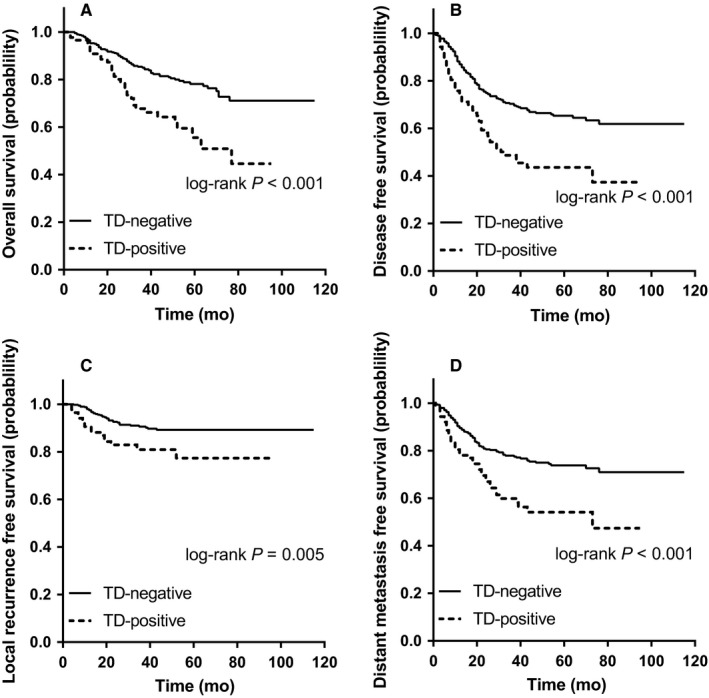
Kaplan‐Meier plots of overall survival (A), disease‐free survival (B), local recurrence‐free survival (C), and distant metastasis‐free survival (D) in all LARC patients stratified by TD subgroups

All LARC patients were separated into TD‐positive and TD‐negative groups. Survival analyses using the Kaplan‐Meier method (Figure 1A‐D) revealed that TD‐positive patients had worse OS, DFS, LRFS, and DMFS compared with TD‐negative patients (OS, *P *<* *0.001; DFS, *P *<* *0.001; LRFS, *P *=* *0.005; DMFS, *P *<* *0.001), indicating that TD was a poor prognostic factor in LARC patients with neo‐CRT.

In addition, univariate Cox survival analyses (Table S2) revealed that OS was significantly associated with cT, cN, interval chemotherapy, operation procedures, differentiation grade, ypT, ypN, number of LNs examined, and neural, vascular, and CRM invasion. DFS was significantly associated with cT, cN, operation procedures, differentiation grade, TRG score, ypN, number of LNs examined, and neural, vascular invasion. In addition, cT, cN, differentiation grade, TRG score, ypN, vascular, and CRM invasion were related to LRFS. Moreover, cN, differentiation grade, ypN, number of LNs examined, and neural, vascular invasion were associated with DMFS.

After adjusting for other clinicopathological factors, multivariate analyses (Table 2) revealed that TD was an independent poor prognostic factor of OS (HR 1.77, 95% CI 1.12‐2.80, *P *=* *0.014), DFS (HR 1.64, 95% CI 1.14‐2.36, *P *=* *0.008) and LRFS (HR 2.07, 95% CI 1.04‐4.13, *P *=* *0.038), but not DMFS (HR 1.43, 95% CI 0.94‐2.19, *P *=* *0.095). In addition, cT, surgical procedures, differentiation grade and number of LNs examined were independent prognostic factors of OS. And cT, surgical procedures and number of LNs examined were independent prognostic factor of DFS. For LRFS, cT, TRG score and CRM remained significant. TRG score and number of LNs examined were independently related to DMFS.

**Table 2:** Multivariate survival analyses of prognostic factors in all LARC patients

Characteristics	OS		DFS		LRFS		DMFS	
	HR^a^ (95% CI)	*P*	HR^a^ (95% CI)	*P*	HR^a^ (95% CI)	*P*	HR^a^ (95% CI)	*P*
cT		0.035		0.029		0.008		0.287
T2	1.00		1.00		1.00		1.00	
T3	0.87 (0.20‐3.76)	0.849	1.80 (0.43‐7.50)	0.422	4524 (< 0.01, > 200)	0.920	2.39 (0.32‐17.58)	0.394
T4	1.61 (0.35‐7.33)	0.539	2.83 (0.66‐12.12)	0.161	12404 (< 0.01, > 200)	0.910	3.18 (0.42‐24.08)	0.263
cN		0.565		0.442		0.368	.	0.728
N0	1.00		1.00		1.00		1.00	
N1	1.45 (0.69‐3.05)	0.334	1.35 (0.78‐2.34)	0.285	1.32 (0.43‐4.06)	0.628	1.08 (0.58‐2.00)	0.805
N2	1.50 (0.70‐3.21)	0.294	1.45 (0.82‐2.56)	0.202	1.95 (0.63‐6.03)	0.247	1.24 (0.66‐2.33)	0.505
Interval chemotherapy		0.662		0.386		0.541	.	0.505
No	1.00		1.00		1.00		1.00	
Yes	0.44 (1.00‐0.44)		0.87 (0.63‐1.19)		1.21 (0.66‐2.24)		0.88 (0.61‐1.28)	
Surgical procedure		0.007		0.001		0.543	.	0.091
APR	1.00		1.00		1.00		1.00	
LAR	0.92 (0.59‐1.45)	0.724	0.85 (0.60‐1.20)	0.355	0.79 (0.42‐1.52)	0.484	0.96 (0.65‐1.41)	0.830
Hartmann	2.61 (1.36‐5.01)	0.004	2.33 (1.36‐3.99)	0.002	1.48 (0.48‐4.53)	0.496	1.98 (1.03‐3.82)	0.041
Differentiation grade		0.001		0.298		0.231	.	0.130
Low	1.00		1.00		1.00	.	1.00	
Middle	0.33 (0.19‐0.57)	< 0.001	0.49 (0.32‐0.77)	0.002	0.45 (0.20‐1.00)	0.051	0.57 (0.34‐0.94)	0.027
High	0.30 (0.07‐1.35)	0.117	0.62 (0.24‐1.57)	0.313	0.69 (0.17‐2.78)	0.598	0.41 (0.12‐1.43)	0.159
Unknown	0.60 (0.34‐1.07)	0.085	0.67 (0.41‐1.07)	0.096	0.49 (0.20‐1.19)	0.114	0.73 (0.42‐1.27)	0.269
TRG		0.558		0.298		0.019	.	0.011
0	1.00		1.00		1.00		1.00	
1	3.88 (0.49‐30.79)	0.200	6.58 (0.68‐63.52)	0.104	2.51 (0.17‐37.54)	0.506	21.48 (3.00‐153.73)	0.002
2	3.22 (0.40‐26.23)	0.274	6.11 (0.62‐60.01)	0.120	1.81 (0.11‐29.05)	0.674	21.31 (2.85‐159.29)	0.003
3	2.86 (0.30‐27.03)	0.360	8.27 (0.79‐86.35)	0.077	6.80 (0.40‐117.02)	0.187	33.56 (4.12‐273.10)	0.001
ypT		0.560		0.698		0.551	.	0.062
T0	1.00		1.00		1.00		1.00	
T1	0.20 (0.02‐2.46)	0.211	0.21 (0.02‐2.36)	0.208	1.61 (0.09‐28.48)	0.746	0.06 (0.01‐0.49)	0.009
T2	0.30 (0.04‐2.50)	0.268	0.25 (0.03‐2.49)	0.238	0.77 (0.05‐12.57)	0.857	0.06 (0.01‐0.44)	0.005
T3	0.40 (0.05‐3.33)	0.396	0.22 (0.02‐2.21)	0.198	0.57 (0.03‐9.72)	0.695	0.06 (0.01‐0.47)	0.007
T4	0.50 (0.05‐4.64)	0.539	0.19 (0.02‐2.11)	0.178	0.33 (0.01‐8.38)	0.503	0.04 (0.004‐0.34)	0.003
ypN		0.242		0.098		0.708	.	0.079
N0	1.00		1.00		1.00		1.00	
N1	1.45 (0.86‐2.45)	0.159	1.52 (1.01‐2.29)	0.043	1.25 (0.57‐2.74)	0.573	0.03 (1.65‐1.05)	0.031
N2	1.68 (0.85‐3.31)	0.135	1.57 (0.91‐2.70)	0.108	1.50 (0.56‐3.99)	0.418	0.11 (1.69‐0.89)	0.107
Tumor Deposits		0.014		0.008		0.038	.	0.095
Negative	1.00		1.00		1.00		1.00	
Positive	1.77 (1.12‐2.80)		1.64 (1.14‐2.36)		2.07 (1.04‐4.13)		1.43 (0.94‐2.19)	
LNs examined		0.003		0.002		0.093	.	0.005
≤11	1.00		1.00		1.00		1.00	
>11	0.52 (0.34‐0.80)		0.60 (0.43‐0.82)		0.60 (0.33‐1.09)		0.58 (0.40‐0.85)	
Vascular invasion		0.807		0.320		0.104	.	0.325
Negative	1.00		1.00		1.00		1.00	
Positive	1.08 (0.57‐2.06)		1.32 (0.77‐2.27)		2.10 (0.86‐5.12)		1.35 (0.74‐2.47)	
Neural invasion		0.925		0.781		0.221	.	0.637
Negative	1.00		1.00		1.00		1.00	
Positive	0.97 (0.55‐1.74)		1.07 (0.67‐1.72)		0.52 (0.18‐1.49)		1.13 (0.67‐1.91)	
CRM invasion		0.068		0.263		0.001	.	0.964
Negative	1.00		1.00		1.00		1.00	
Positive	4.21 (0.90‐19.78)		2.32 (0.53‐10.16)		19.50 (3.46‐110.02)		< 0.01 (<0.01, >200)	

HR, hazard ratio; TD, tumor deposit; LN, lymph node; TRG, tumor regression grade; CRM, circumferential resection margin.

a Multivariate Cox regression model controlling for cT, cN, interval chemotherapy, surgical procedure, tumor grade, TRG score, ypT, ypN, No. of LN examined, vascular invasion, neural invasion, and CRM.

### 3.3 Prognostic value of tumor deposits in LN‐negative patients

**Figure 2:**
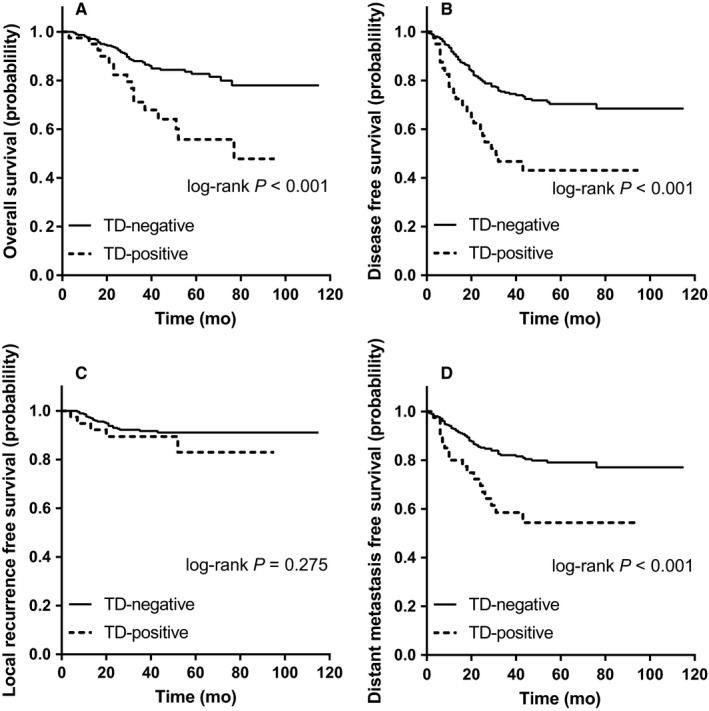
Kaplan‐Meier plots of overall survival (A), disease‐free survival (B), local recurrence‐free survival (C), and distant metastasis‐free survival (D) in LN‐negative LARC patients stratified by TD subgroups

Tumor deposit may exhibit different prognostic values in LN‐negative and LN‐positive patients. Thus, we investigated the prognostic value of TDs in the two LN subgroups. For LN‐negative patients, survival analyses using the Kaplan‐Meier method (Figure 2A‐D) revealed poor OS, DFS, and DMFS in TD‐positive patients compared with TD‐negative patients (OS, *P *<* *0.001; DFS, *P *<* *0.001; LRFS, *P *=* *0.275; DMFS, *P *<* *0.001) but not LRFS (*P *=* *0.275). Significant clinicopathological factors (*P *<* *0.1) for OS, DFS, LRFS, and DMFS in univariate Cox models were included in multivariate Cox survival analyses. The results are presented in Table S3. In LN‐negative patients, TD remained a poor prognostic factor of OS (OS, HR 2.52, 95% CI 1.27‐5.02, *P *=* *0.008), DFS (HR 2.46, 95% CI 1.47‐4.12, *P *=* *0.001), and DMFS (HR 2.34, 95% CI 1.29‐4.25, *P *=* *0.005) independent of other variables, not LRFS (HR 2.11, 95% CI 0.70‐6.34, *P *=* *0.184).

### 3.4 Prognostic value of tumor deposits in LN‐positive patients

However, for LN‐positive patients, survival analyses using the Kaplan‐Meier method (Figure S1a‐d) revealed no association between TD groups and prognosis (OS, *P *=* *0.382; DFS, *P *=* *0.389; LRFS, *P *=* *0.075; DMFS, *P *=* *0.700).

Of note, the number of positive LNs varied from 1 to 15, exhibiting great heterogeneity in survival time. Thus, we examined the prognostic value of TD in patients with a specific number of positive LNs, such as 1, 2, 3, and greater than 3. As shown in Table S4, significant prognostic differences were exclusively identified in patients with one positive LN and poor OS and LRFS in TD‐positive patients (OS, *P *=* *0.010; LRFS, *P *=* *0.013). However, for patients with greater than 1 positive LNs, no difference in OS, DFS, LRFS, and DMFS was noted between the two TD groups.

What's more, we combined patients with negative LN and patients with only one positive LN together, then performed multivariate analyses again. We found that TD was associated with poor OS (HR 2.53, 95% CI 1.43‐4.47, *P *=* *0.001), DFS (HR 2.03, 95% CI 1.29‐3.19, *P *=* *0.002), LRFS (HR 2.77, 95% CI 1.09‐7.03, *P *=* *0.032) and DMFS (HR 1.81, 95% CI 1.07‐3.07, *P *=* *0.027) in patients with no more than one positive LN, independent of other clinicopathological features. It indicated that TD was an independent poor prognostic factor in LARC patients following neo‐CRT, especially in patients with no more than one positive LN.

### 3.5 The relevance of tumor deposit count and prognosis

The abovementioned findings revealed the poor prognostic value of TD in LARC patients with neo‐CRT. However, the relevance of the TD count and prognosis was unclear. Thus, we defined four grades of TD counts according to the criteria presented in Table S5 and classified the patients into four groups. After replacing the variable “TD status” by the new variable “TD grade”, similar Kaplan‐Meier and multivariate Cox survival analyses (Table S6) were performed again to examine the prognostic value of TD numbers.

For all patients, although the *P*‐values in Kaplan‐Meier survival analyses were all < 0.05 in OS, DFS, LRFS, and DMFS, the HR‐values did not increase successively among grade 1 vs 0, grade 2 vs 0, and grade 3 vs 0 in multivariate analyses. For LN‐positive patients, Kaplan‐Meier survival analyses revealed survival differences only in LRFS (*P *=* *0.012), and multivariate analyses did not reveal increasing HR values for LRFS.

However, multivariate analyses in LN‐negative patients exhibited successively increasing HR values among grade 1 vs 0, grade 2 vs 0, and grade 3 vs 0 only in terms of OS and DFS. This finding indicated that the increasing TD counts were associated with decreasing survival only in LN‐negative patients, not in LN‐positive patients.

### 3.6 Staging value of tumor deposits

We used three methods of N categorization (oN, n1N, and n2N) to evaluate the impacts of TD count on N staging method. The changes in patient distributions among the three methods are presented in Table S7. On comparing the n1N and oN methods, 40 patients changed from N0 to N1c. These 40 patients were then transformed into 8 N1a, 10 N1b, 13 N2a, and 9 N2b patients using the n2N method. Finally, for a total of 32 patients, N1 categorization changed to N2 using the n2N method.

After replacing the variables “TD status, ypN” by the new variables “n1N” or “n2N”, we independently performed similar multivariate Cox survival analyses twice. The results of survival analyses using the three methods are presented in Table 3. Of note, after adjusting for multiple relevant variables, n1N and n2N were both independent prognostic factors of OS (n1N, *P *=* *0.003; n2N, *P *=* *0.003), DFS (n1N, *P *<* *0.001; n2N, *P *<* *0.001), and DMFS (n1N, *P *=* *0.001; n2N, *P *=* *0.001) but not LRFS (n1N, *P *=* *0.202; n2N, *P *=* *0.079). However, oN was not independently associated with OS, DFS, LRFS, and DMFS. These findings indicated that TD had an important impact on prognosis and should be taken into consideration when performing N staging.

**Table 3:** Adjusted HRs and 95% CIs for survival by three N staging methods in all LARC patients

Method	OS		DFS		LRFS		DMFS	
	HR^a^ (95% CI)	*P*	HR^a^ (95% CI)	*P*	HR^a^ (95% CI)	*P*	HR^a^ (95% CI)	*P*
oN		0.242		0.098		0.708		0.079
N0	1.00		1.00		1.00		1.00	
N1	1.45 (0.86‐2.45)	0.159	1.52 (1.01‐2.29)	0.043	1.25 (0.57‐2.74)	0.573	1.03 (1.65‐1.05)	0.031
N2	1.68 (0.85‐3.31)	0.135	1.57 (0.91‐2.70)	0.108	1.50 (0.56‐3.99)	0.418	1.11 (1.69‐0.89)	0.107
n1N		0.003		<0.001		0.202		0.001
N0	1.00		1.00		1.00		1.00	
N1	2.24 (1.36‐3.69)	0.002	2.18 (1.50‐3.17)	<0.001	1.63 (0.79‐3.39)	0.190	2.16 (1.40‐3.33)	<0.001
N2	2.81 (1.38‐5.72)	0.004	2.40 (1.39‐4.14)	0.002	2.35 (0.90‐6.19)	0.083	2.40 (1.27‐4.53)	0.007
n2N		0.003		<0.001		0.079		0.001
N0	1.00		1.00		1.00		1.00	
N1	2.14 (1.27‐3.60)	0.004	2.06 (1.39‐3.04)	<0.001	1.45 (0.67‐3.12)	0.342	2.10 (1.34‐3.28)	0.001
N2	2.79 (1.50‐5.19)	0.001	2.65 (1.65‐4.26)	<0.001	2.72 (1.13‐6.57)	0.026	2.48 (1.42‐4.32)	0.001

HR, hazard ratio; OS, overall survival; DFS, disease‐free survival; LRFS, local recurrence‐free survival; DMFS, distant metastasis‐free survival.

a Multivariate Cox regression model controlling for cT, cN, interval chemotherapy, surgical procedure, tumor grade, TRG score, ypT, ypN (using three N staging methods), No. of LN examined, vascular invasion, neural invasion, and CRM.

**Figure 3:**
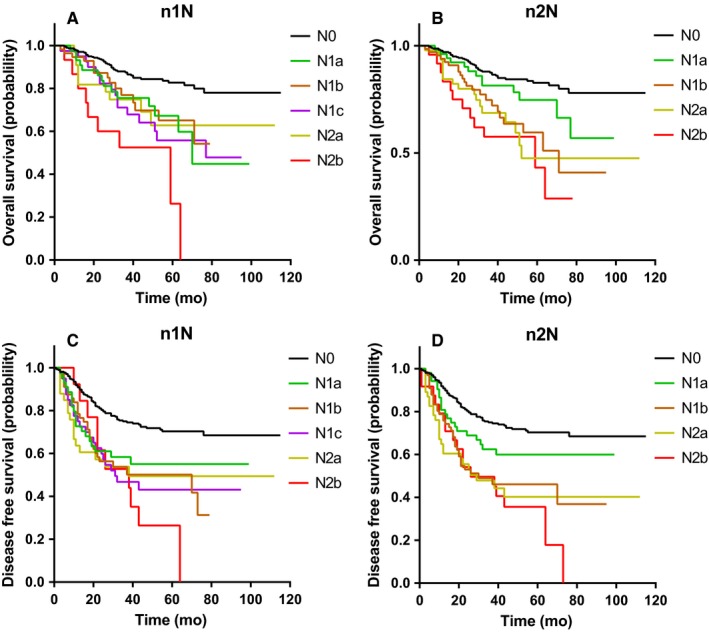
Comparison of overall survival (A, B), disease‐free survival (C, D) in all LARC patients stratified by n1N and n2N staging methods

To compare the efficacy of n1N and n2N methods, we performed survival analyses using the Kaplan‐Meier method, illustrating the prognostic differences among multiple N subgroups. The n1N method could distinguish N0 patients from N1a‐2b patients in OS, DFS, DMFS, and LRFS. However, as shown in Figure 3A‐D, the n2N method could distinguish N0 as well as N1a patients from N1b‐2b patients in OS and DFS. Similar trend was also found in DMFS (Figure S2C,D). Although no difference in LRFS was noted between N0 and N1a patients using the n2N method, the method could distinguish N0‐1a patients from N1b‐2b patients (Figure S2a,b). Thus, it seemed that the n2N method exhibited better prognostic efficacy compared with the n1N method. Thus, it was probably better to consider one TD as one positive LN.

### 3.7 The relevance of one tumor deposit and one positive lymph node

To investigate whether one TD was equivalent to one positive LN, we compared the survival difference between the following two groups of patients using the Kaplan‐Meier method: LN‐negative patients with TDs (its positive number was *i*) and TD‐negative patients with positive LNs (its positive number was *j*). Interestingly, when the positive number equaled one (*i* = *j* = 1) and two (*i* = *j* = 2), no survival difference was noted between the two groups (all *P *>* *0.1) (Table S8). The results indicated that one TD likely had the same poor prognostic value as one positive LN.

## 4 DISCUSSION

Tumor deposits are typically found in the perirectal and mesenteric adipose tissue around rectal adenocarcinomas. Several editions of the American Joint Committee on Cancer (AJCC) staging manual have defined TDs. Gabriel et al defined TD in 1935 for the first time and considered the invasion depth of TD‐positive patients as pT3. In 1997, the fifth edition of the AJCC staging manual defined TD according to the criterion of maximum diameter. Nodules ≤3 mm were considered as TDs, and nodules >3 mm were considered as positive LNs. In 2002, the sixth edition of the AJCC staging manual defined TDs according to criterion of their contours. Nodules with irregular contours were considered as TDs, and nodules with regular contours were considered as positive LNs.^9^ Furthermore, in 2009, the definition of TD changed considerably in the seventh edition of the AJCC staging manual. TDs should be located in the pericolorectal fat or adjacent mesocolic fat, away from the leading edge of the tumor, with no evidence of residual lymph node tissue, and within the lymph drainage area of the primary carcinoma.^10^ The current eighth edition of the AJCC staging manual emphasizes the importance of no evidence of residual LN, neural, and vascular tissues with other details remaining unchanged. According to the seventh and eighth editions of AJCC stating manuals, patients with positive TDs but negative LNs are classified as N1c. However, TD is not included in the N category for patients with positive LNs.

Previously, several studies reported that TDs were associated with poor prognosis in colorectal cancers. Ueno et al found that TD was a poor prognostic factor independent of pT and pN.^11^, ^12^, ^13^ Tong et al reported that TD‐positive patients had worse survival compared with TD‐negative patients among LN‐negative patients.^14^ Nagayoshi et al verified the independent prognostic value of TD not only in LN‐negative patients but also in patients with less than four positive LNs.^15^ These findings indicated that patients with TDs should probably receive more aggressive chemotherapy to control local recurrence and distant metastasis.

Song et al studied 513 patients with stage III colorectal cancers to evaluate the impact of TD on tumor staging.^16^ They classified the patients using the following two staging methods: method 1 (pN, pTNM) based on the seventh edition of AJCC staging manual and method 2 (npN, npTNM), which considered one TD as one positive LN. Univariate and multivariate survival analyses revealed pN, pTNM, npN, and npTNM were all associated with prognosis. However, npN and nTNM exhibited improved predictive ability of the prognosis compared with pN and nTNM by calculating the Harrell's C index. In addition, TD‐negative and TD‐positive patients did not exhibit significant survival differences within the same subgroup of npN, indicating that one TD may have equal prognostic value to one positive LN.

However, patients recruited in these studies did not receive neo‐CRT. Some changes will occur after CRT, such as decreased number of TDs, degenerative tumor cells, the formation of mucous lakes, and tissue fibrosis.^7^ These changes may influence the accuracy of the prognostic and staging values of TD in patients with neo‐CRT.

Several studies have assessed LARC patients with neo‐CRT, but the conclusions were controversial. In 2011, Song et al retrospectively analyzed 136 pT3N0M0 patients with neo‐CRT and did not identify any significant differences in OS and DFS between TD‐positive and TD‐negative patients.^17^ Thus, in their opinion, the N1c category was not applicable to patients with neo‐CRT. However, in 2014, Gopal et al reported that TD‐positive patients exhibited increased frequencies of metastatic LNs (*P *=* *0.035), distant metastasis (*P *=* *0.006), and worse survival (*P *=* *0.027) after analyzing 110 patients with neo‐CRT.^18^ In 2015, another study with 310 LARC patients was performed by Zhang et al.^19^ Survival analyses using the Kaplan‐Meier method revealed worse OS, DFS, and DMFS in TD‐positive patients, not LRFS. Multivariate survival analyses revealed that TD remained an independent poor prognostic factor of OS and DFS (OS, HR 2.44, *P *=* *0.004; DFS, HR 1.99, *P *=* *0.007). In TD‐positive patients, patients with chemotherapy after operation exhibited significantly better OS (*P *=* *0.045) and DMFS (*P *=* *0.026) compared with those without chemotherapy. These findings indicated that TD‐positive patients likely needed more aggressive chemotherapy to acquire longer survival despite receiving neo‐CRT. In 2016, Wei et al analyzed 4813 LARC patients with neo‐CRT from the Surveillance, Epidemiology, and End Results (SEER) database and verified the poor independent prognostic value of TD in cancer‐specific survival (CSS, HR 2.25, *P *<* *0.001).^20^ In addition, the CSS of N1c patients was significantly worse compared with N0 patients, confirming the application of the N1c category in LARC patients with neo‐CRT. Recently, Bouquot et al included 1122 colorectal patients to evaluate the impact of N1c category in both colon and rectal cancers.^21^ N1c category were identified only in 57 patients (24 colon cancers and 33 rectal cancers) and was associated with rectal cancer, advanced tumor stage (~90% T3‐T4), and synchronous metastases (40%). A total of 33 N1c nonmetastatic colon (14 patients) and rectal tumors (19 patients, of which 13 patients received neo‐CRT) were matched to 161 N0, N1a, and N1b colorectal specimens. There was no difference in terms of 3‐year OS among N0, N1a, N1b, and N1c tumors (*P *=* *0.9633). DFS was significantly worse for N1c tumors compared to N0 tumors (*P *=* *0.017), but not significantly different among N1a, N1b, and N1c tumors (*P *=* *0.0363). However, difference in DFS between N1c and N0 was significant only for colon cancers (*P *=* *0.014), not rectal cancer (*P *=* *0.253), which indicate the difficulty of categorizing N1c in rectal cancer after neoadjuvant therapy. At present, there is no data to classify TD after neoadjuvant therapy in a best way.

In the present study, we retrospectively analyzed 495 LARC patients after neo‐CRT to evaluate the prognostic significance of TDs, verified the applicability of the N1c category in those tumors, and explored the appropriate methods of N staging for those patients for the first time.

First, the patients were separated into two groups: TD‐positive group and TD‐negative group. The chi‐square tests revealed that TD‐positive LARC patients with neo‐CRT tended to exhibit worse malignant clinicopathological features, which were similar to those without neo‐CRT. Second, survival analyses were performed. In multivariate analysis, TD was an independent poor prognostic factor of OS, DFS, and LRFS in all patients. In LN‐negative patients, TD was also an independent poor prognostic factor of OS, DFS, and DMFS. The N1c category described in eighth edition of AJCC staging manual is also applicable in LN‐negative patients, which was in agreement with the findings by Wei et al.^20^ However, it was not consistent with results by Bouquot et al, which showed worse survival of N1c category compared with N0 category only in colon cancers, not rectal cancers.^21^ The reason may be the small sample size of N1c rectal cancers (19 patients), including only 13 rectal cancers receiving neo‐CRT.

Interestingly, in LN‐positive patients, we found that TD was associated with poor OS and LRFS only in patients with one positive LN. Moreover, TD was associated with poor OS, DFS, LRFS, and DMFS in patients with no more than one positive LN, independent of other clinicopathological features. For patients with greater than two positive LNs, no differences in OS, DFS, LRFS, and DMFS were noted between the two TD groups. In patients with several positive LNs, the key factor that has an essential impact on local recurrence and distant metastasis is positive LNs, not positive TDs. The patients in the two TD groups both have relatively bad survival regardless of their TD status.

Then, we analyzed the associations between TD count and survival. Multivariate analyses revealed that successively increasing HR values among grade 1 vs 0, grade 2 vs 0, and grade 3 vs 0 exclusively existed in LN‐negative patients in terms of OS and DFS. Increasing TD count was associated with decreasing survival exclusively in LN‐negative patients but not in all patients or LN‐positive patients. These findings indicated that the poor prognostic value of TD is more obvious and significant in LN‐negative patients. It is the positive LNs that account more for the bad prognosis in LN‐positive patients.

Finally, three independent multivariate analyses according to the three N staging methods were performed. Our results demonstrated that oN was not an independent prognostic factor, whereas n1N and n2N were independent poor prognostic factors of OS, DFS, and DMFS but not LRFS. The first multivariate survival analysis including the method 1 (oN) showed that oN was not an independent prognostic factor. In other words, in LARC patients with neo‐CRT, N staging method not including TDs was not powerful enough to predict survivals. The second multivariate survival analysis including the method 2 (n1N) showed that n1N was an independent poor prognostic factor of OS, DFS, and DMFS. It showed that N1c category was necessary for LARC patients with neo‐CRT, which would improve the efficacy of N staging. The third multivariate survival analysis including the method 3 (n2N) showed that n2N was also an independent poor prognostic factor of OS, DFS, and DMFS. Considering one TD as one positive LN could also improve the efficacy of N staging. Furthermore, Kaplan‐Meier plots comparing n1N method with n2N method revealed that the prognostic efficacy of the n2N method seemed to be better than the n1N method. Thus, it was probably better to consider one TD as one positive LN and include the total number of metastatic LNs and TDs into N staging, not just considering those LN‐negative patients with TDs as N1c category. In addition, no statistical differences in OS, DFS, LRFS, and DMFS were noted between TD(+)LN(‐) patients and LN(+)TD(‐) patients with the same counts of TDs and positive LNs, which verified the equal value of one positive TD and one positive LN.

However, the conclusion regarding the TD staging value should be interpreted with caution. Only 17.8% (88 of 495) patients were TD positive. The number of TD‐positive patients was relatively small (even only several patients in one subgroup) when they were separated into different LN subgroups or N stage subgroups. The sample size was not sufficient to analyze the accurate staging value of TD, which was the main limitation of this study. We can only conclude that TD should be taken into consideration when performing N staging. In addition, although we can advise that the N1c category should be included for TD‐positive LARC patients with negative LNs, which is the same as those without neo‐CRT, however, given the small number of TD‐positive patients, we cannot assess whether an extra N category for TD‐positive LARC patients with only one positive LN is necessary.

In this study, we only used Kaplan‐Meier analyses to compare the prognostic efficacy between nN1 and nN2 staging methods and to compare the prognostic value between one positive TD and one positive LN. The statistical methods were relatively simple, and advanced methods, such as R language, are needed. Thus, further studies with larger samples and advanced statistical methods are needed to investigate the accurate staging value of TD and whether one positive TD should have the same prognostic value as one positive LN. Future studies should develop an ideal staging model that includes not only T, N, and M categories but also extra categories characterized by positive TDs with different LN statuses.

In summary, TD is an independent poor prognostic factor in LARC patients following neo‐CRT, especially in patients with no more than one positive LN. The N1c category of eighth edition of the AJCC staging manual is also applicable in LN‐negative patients. When counting the total number of metastatic LNs and TDs, the new N staging method (n2N) can achieve a better efficacy than others. Of course, further studies with large samples are needed to investigate the accurate staging value of TD and whether one TD could be considered as one positive LN.

## CONFLICT OF INTEREST

The authors have no conflict of interest.
