# Supplementary material for: Poor prognostic and staging value of tumor deposit in locally advanced rectal cancer with neoadjuvant chemoradiotherapy
Source: Cancer Med. 2019 Feb 21;8(4):1508–20. doi: 10.1002/cam4.2034 (PMC6488131; doi:10.1002/cam4.2034)
Supplement: Supplementary file 3 [file CAM4-8-1508-s003.docx]

**Appendix**

**Supplementary Tables**

**Table S1.** Definition of three N staging methods (oN, n1N and n2N)

| N stages | N subgroups | oN method | n1N method | n2N method |
| --- | --- | --- | --- | --- |
| N0 | N0 | 0 | 0 | 0 |
| N1 | N1a | 1 | 1 | 1 |
|  | N1b | 2-3 | 2-3 | 2-3 |
|  | N1c | --- | TD+LN- | --- |
| N2 | N2a | 4-6 | 4-6 | 4-6 |
|  | N2b | ≥ 7 | ≥ 7 | ≥ 7 |

Abbreviations: TD, tumor deposit; LN, lymph node; AJCC, American Joint Committee on Cancer.

**Table S2.** Univariate survival analyses of prognostic factors in all LARC patients

| Characteristics | OS | | DFS | | LRFS | | DMFS | |
| --- | --- | --- | --- | --- | --- | --- | --- | --- |
|  | HR^#^ 95% CI | P | HR^#^ 95% CI | P | HR^#^ 95% CI | P | HR^#^ 95% CI | P |
| Age (y) |  | 0.671 |  | 0.503 |  | 0.340 |  | 0.441 |
| < 50 | 1.00 |  | 1.00 |  | 1.00 |  | 1.00 |  |
| ≥ 50 | 0.92 (0.62-1.36) |  | 1.11 (0.81-1.53) |  | 0.77 (0.44-1.33) |  | 1.16 (0.80-1.67) |  |
| Gender |  | 0.244 |  | 0.623 |  | 0.750 |  | 0.464 |
| Male | 1.00 |  | 1.00 |  | 1.00 |  | 1.00 |  |
| Female | 0.77 (0.49-1.20) |  | 1.09 (0.79-1.50) |  | 0.91 (0.49-1.67) |  | 1.15 (0.79-1.66) |  |
| cT |  | 0.041 |  | 0.050 |  | 0.028 |  | 0.343 |
| T2 | 1.00 |  | 1.00 |  | 1.00 |  | 1.00 |  |
| T3 | 0.97 (0.24-3.94) | 0.963 | 1.88 (0.47-7.61) | 0.374 | 7596  (< 0.001, > 200) | 0.920 | 2.87 (0.40-20.60) | 0.294 |
| T4 | 1.69 (0.40-7.09) | 0.475 | 2.78 (0.67-11.50) | 0.157 | 16708  (< 0.001, > 200) | 0.914 | 3.53 (0.48-25.98) | 0.215 |
| cN |  | 0.009 |  | 0.009 |  | 0.020 |  | 0.014 |
| N0 | 1.00 |  | 1.00 |  | 1.00 |  | 1.00 |  |
| N1 | 1.36 (0.68-2.73) | 0.384 | 1.30 (0.77-2.22) | 0.327 | 1.29 (0.43-3.83) | 0.647 | 1.07 (0.59-1.94) | 0.830 |
| N2 | 2.26 (1.15-4.43) | 0.018 | 1.92 (1.14-3.22) | 0.014 | 2.67 (0.94-7.55) | 0.064 | 1.75 (0.98-3.11) | 0.057 |
| Distance from anus (cm) |  | 0.346 |  | 0.829 |  | 0.457 |  | 0.834 |
| ≤ 5 | 1.00 |  | 1.00 |  | 1.00 |  | 1.00 |  |
| > 5 | 1.20 (0.82-1.75) |  | 1.03 (0.77-1.39) |  | 0.81 (0.46-1.42) |  | 1.04 (0.74-1.47) |  |
| Radiation dose (Gy) |  | 0.842 |  | 0.614 |  | 0.943 |  | 0.382 |
| ≤ 50 | 1.00 |  | 1.00 |  | 1.00 |  | 1.00 |  |
| > 50 | 0.96 (0.61-1.49) |  | 0.91 (0.63-1.31) |  | 0.98 (0.50-1.90) |  | 0.82 (0.53-1.27) |  |
| Interval chemotherapy |  | 0.002 |  | 0.067 |  | 0.671 |  | 0.175 |
| No | 1.00 |  | 1.00 |  | 1.00 |  | 1.00 |  |
| Yes | 0.56 (0.38-0.81) |  | 0.76 (0.56-1.02) |  | 1.13 (0.64-2.00) |  | 0.79 (0.56-1.11) |  |
| Surgical procedure |  | 0.001 |  | 0.001 |  | 0.302 |  | 0.084 |
| APR | 1.00 |  | 1.00 |  | 1.00 |  | 1.00 |  |
| LAR | 0.94 (0.62-1.42) | 0.771 | 0.83 (0.60-1.15) | 0.264 | 0.67 (0.37-1.22) | 0.187 | 0.90 (0.63-1.30) | 0.576 |
| Hartmann | 2.80 (1.56-5.03) | 0.001 | 2.24 (1.37-3.66) | 0.001 | 1.34 (0.47-3.77) | 0.586 | 1.85 (1.00-3.40) | 0.049 |
| Adjuvant chemotherapy; |  | 0.355 |  | 0.442 |  | 0.364 |  | 0.634 |
| No | 1.00 |  | 1.00 |  | 1.00 |  | 1.00 |  |
| Yes | 1.53 (0.62-3.75) |  | 1.28 (0.68-2.43) |  | 1.92 (0.47-7.90) |  | 1.19 (0.58-2.43) |  |
| Differentiation grade |  | < 0.001 |  | < 0.001 |  | 0.003 |  | 0.003 |
| Low | 1.00 |  | 1.00 |  | 1.00 | . | 1.00 |  |
| Middle | 0.27 (0.16-0.44) | .000 | 0.39 (0.26-0.58) | < 0.001 | 0.34 (0.17-0.67) | 0.002 | 0.46 (0.29-0.73) | 0.001 |
| High | 0.20 (0.05-0.85) | .029 | 0.48 (0.20-1.14) | 0.095 | 0.74 (0.21-2.55) | 0.629 | 0.34 (0.10-1.11) | 0.073 |
| Unknown | 0.41 (0.26-0.64) | < 0.001 | 0.44 (0.30-0.64) | < 0.001 | 0.31 (0.16-0.63) | 0.001 | 0.48 (0.30-0.76) | 0.002 |
| TRG |  | 0.247 |  | 0.035 |  | 0.009 |  | 0.103 |
| 0 | 1.00 |  | 1.00 |  | 1.00 |  | 1.00 |  |
| 1 | 1.26 (0.71-2.25) | 0.431 | 1.65 (1.04-2.64) | 0.036 | 2.41 (0.95-6.12) | 0.064 | 1.48 (0.86-2.54) | 0.153 |
| 2 | 1.66 (0.98-2.79) | 0.058 | 1.86 (1.21-2.86) | 0.005 | 1.91 (0.77-4.73) | 0.164 | 1.72 (1.05-2.82) | 0.031 |
| 3 | 1.21 (0.48-3.03) | 0.684 | 2.04 (1.07-3.89) | 0.031 | 5.44 (1.93-15.30) | 0.001 | 2.18 (1.07-4.44) | 0.031 |
| ypT |  | 0.026 |  | 0.065 |  | 0.240 |  | 0.072 |
| T0 | 1.00 |  | 1.00 |  | 1.00 |  | 1.00 |  |
| T1 | 0.57 (0.13-2.44) | 0.448 | 1.16 (0.48-2.81) | 0.744 | 3.61 (1.02-12.79) | 0.047 | 0.95 (0.33-2.76) | 0.928 |
| T2 | 0.94 (0.51-1.73) | 0.851 | 1.51 (0.94-2.42) | 0.086 | 1.99 (0.77-5.12) | 0.155 | 1.20 (0.70-2.05) | 0.514 |
| T3 | 1.68 (1.00-2.83) | 0.052 | 1.87 (1.22-2.89) | 0.005 | 2.46 (1.01-5.97) | 0.047 | 1.81 (1.12-2.93) | 0.016 |
| T4 | 2.19 (1.02-4.71) | 0.046 | 1.72 (0.85-3.47) | 0.130 | 1.39 (0.28-6.86) | 0.690 | 1.13 (0.46-2.80) | 0.786 |
| ypN |  | < 0.001 |  | < 0.001 |  | 0.009 |  | < 0.001 |
| N0 | 1.00 |  | 1.00 |  | 1.00 |  | 1.00 |  |
| N1 | 1.89 (1.21-2.95) | 0.005 | 1.92 (1.36-2.71) | < 0.001 | 1.83 (0.97-3.46) | 0.064 | 2.13 (1.44-3.15) | < 0.001 |
| N2 | 2.82 (1.68-4.73) | < 0.001 | 2.53 (1.66-3.84) | < 0.001 | 2.90 (1.41-5.95) | 0.004 | 2.58 (1.59-4.18) | < 0.001 |
| Tumor Deposits |  | < 0.001 |  | < 0.001 |  | 0.006 |  | < 0.001 |
| negative | 1.00 |  | 1.00 |  | 1.00 |  | 1.00 |  |
| positive | 2.23 (1.48-3.35) |  | 2.06 (1.48-2.87) |  | 2.26 (1.26-4.07) |  | 2.04 (1.40-2.98) |  |
| LNs examined |  | 0.030 |  | 0.012 |  | 0.293 |  | 0.011 |
| ≤ 11 | 1.00 |  | 1.00 |  | 1.00 |  | 1.00 |  |
| > 11 | 0.64 (0.43-0.96) |  | 0.67 (0.49-0.92) |  | 0.74 (0.42-1.30) |  | 0.63 (0.44-0.90) |  |
| Vascular invasion |  | 0.002 |  | < 0.001 |  | 0.001 |  | < 0.001 |
| negative | 1.00 |  | 1.00 |  | 1.00 |  | 1.00 |  |
| positive | 2.42 (1.40-4.17) |  | 2.47 (1.58-3.85) |  | 3.39 (1.66-6.95) |  | 2.65 (1.61-4.36) |  |
| Neural invasion |  | 0.058 |  | 0.036 |  | 0.830 |  | 0.034 |
| negative | 1.00 |  | 1.00 |  | 1.00 |  | 1.00 |  |
| positive | 1.63 (0.98-2.70) |  | 1.56 (1.03-2.37) |  | 0.90 (0.36-2.27) |  | 1.65 (1.04-2.64) |  |
| CRM invasion |  | 0.025 |  | 0.200 |  | 0.002 |  | 0.607 |
| negative | 1.00 |  | 1.00 |  | 1.00 |  | 1.00 |  |
| positive | 4.94 (1.22-20.06) |  | 2.49 (0.62-10.04) |  | 9.61 (2.34-39.51) |  | 0.05 (< 0.001, 4620) |  |

Note: ^#^ All HRs (95% CI) were calculated through univariate Cox regression models.

**Table S3.** Multivariate survival analyses of prognostic factors in LN-negative LARC patients

| Characteristics | OS | | DFS | | | | LFRS | | DMFS | |
| --- | --- | --- | --- | --- | --- | --- | --- | --- | --- | --- |
|  | HR^#^ (95% CI) | P | HR^#^ (95% CI) | | | P | HR^#^ (95% CI) | P | HR^#^ (95% CI) | P |
| cT |  | 0.004 |  | | | 0.002 |  | 0.001 |  | 0.117 |
| T2 | 1.00 |  | 1.00 | | |  | 1.00 |  | 1.00 |  |
| T3 | 0.88 (0.11-7.29) | 0.903 | 2.24 (0.30-17.07) | | | 0.435 | 2794  (< 0.01, > 200) | 0.922 | 4453  (< 0.01, > 200) | 0.884 |
| T4 | 2.51 (0.29-21.43) | 0.401 | 4.88 (0.63-37.60) | | | 0.128 | 13593  (< 0.01, > 200) | 0.907 | 7917  (< 0.01, > 200) | 0.876 |
| cN |  | 0.399 |  | | | 0.793 |  | 0.258 |  | 0.807 |
| N0 | 1.00 |  | 1.00 | | |  | 1.00 |  | 1.00 |  |
| N1 | 1.70 (0.74-3.90) | 0.211 | 1.18 (0.65-2.12) | | | 0.588 | 1.23 (0.38-4.02) | 0.730 | 0.88 (0.45-1.71) | 0.700 |
| N2 | 1.24 (0.52-2.95) | 0.621 | 1.23 (0.67-2.28) | | | 0.501 | 2.38 (0.70-8.17) | 0.167 | 1.05 (0.53-2.09) | 0.888 |
| Interval chemotherapy |  | 0.081 |  | | | 0.777 |  | 0.233 |  | 0.796 |
| No | 1.00 |  | 1.00 | | |  | 1.00 |  | 1.00 |  |
| Yes | 0.61 (0.35-1.06) |  | 1.07 (0.69-1.65) | | |  | 1.84 (0.68-5.01) |  | 1.07 (0.641-1.79) |  |
| Surgical procedure |  | 0.003 |  | | | .040 |  | 0.060 |  | 0.440 |
| APR | 1.00 |  | 1.00 | | |  | 1.00 |  | 1.00 |  |
| AR | 0.72 (0.39-1.34) | 0.305 | 0.89 (0.57-1.40) | | | 0.622 | 0.52 (0.20-1.33) | 0.173 | 1.03 (0.62-1.72) | 0.911 |
| Hartmann | 3.60 (1.49-8.70) | 0.005 | 2.34 (1.12-4.89) | | | 0.024 | 2.98 (0.80-11.13) | 0.104 | 1.81 (0.72-4.55) | 0.206 |
| Differentiation grade |  | 0.066 |  | | | 0.192 |  | 0.098 |  | 0.816 |
| Low | 1.00 |  | 1.00 | | |  | 1.00 | . | 1.00 |  |
| Middle | 0.33 (0.15-0.74) | 0.007 | 0.48 (0.24-0.96) | | | 0.036 | 0.28 (0.08-0.90) | 0.033 | 0.75 (0.31-1.80) | 0.517 |
| High | < 0.001  (< 0.01, > 200) | 0.971 | 0.75 (0.19-2.96) | | | 0.676 | 0.75 (0.11-5.03) | 0.769 | 0.45 (0.05-4.03) | 0.478 |
| Unknown | 0.47 (0.20-1.11) | 0.085 | 0.63 (0.31-1.31) | | | 0.219 | 0.28 (0.08-1.00) | 0.051 | 0.91 (0.36-2.31) | 0.841 |
| TRG |  | 0.018 |  | | | 0.006 |  | 0.162 |  | 0.038 |
| 0 | 1.00 |  | 1.00 | | |  | 1.00 |  | 1.00 |  |
| 1 | 13.34 (1.78-100.1) | 0.012 | 21.58 (3.07-151.8) | | | 0.002 | 2.28 (0.08-64.74) | 0.629 | 17.38 (2.21-136.8) | 0.007 |
| 2 | 6.45 (0.79-52.47) | 0.081 | 13.93 (1.86-104.3) | | | 0.010 | 0.82 (0.03-26.23) | 0.909 | 13.73 (1.61-117.3) | 0.017 |
| 3 | 4.62 (0.37-58.08) | 0.236 | 8.76 (0.93-82.73) | | | 0.058 | 3.27 (0.09-119.5) | 0.519 | 8.82 (0.77-100.8) | 0.080 |
| ypT |  | 0.108 |  |  |  | 0.982 |  | 0.133 |  |  |
| T0 | 1.00 |  | 1.00 |  | 1.00 |  | 1.00 |  |  |  |
| T1 | 0.07 (0.01-1.14) | 0.062 | 0.06 (0.01-0.54) | | | 0.012 | 1.60 (0.04-60.01) | 0.800 | 0.03 (0.002-0.49) | 0.014 |
| T2 | 0.11 (0.01-0.81) | 0.030 | 0.10 (0.01-0.68) | | | 0.019 | 1.33 (0.04-41.33) | 0.870 | 0.08 (0.01-0.65) | 0.018 |
| T3 | 0.22 (0.03-1.67) | 0.144 | 0.11 (0.02-0.82) | | | 0.031 | 0.99 (0.03-32.83) | 0.995 | 0.10 (0.01-0.82) | 0.032 |
| T4 | 0.14 (0.01-1.41) | 0.094 | 0.09 (0.01-0.78) | | | 0.029 | < 0.001  (< 0.01, > 200) | 0.838 | 0.10 (0.01-1.01) | 0.051 |
| TD |  | 0.008 |  | | | 0.001 |  | 0.184 |  | 0.005 |
| Negative | 1.00 |  | 1.00 | | |  | 1.00 |  | 1.00 |  |
| Positive | 2.52 (1.27-5.02) |  | 2.46 (1.47-4.12) | | |  | 2.11 (0.70-6.34) |  | 2.34 (1.29-4.25) |  |
| LNs examined |  | 0.120 |  | | | 0.132 |  | 0.498 |  | 0.293 |
| ≤ 11 | 1.00 |  | 1.00 | | |  | 1.00 |  | 1.00 |  |
| > 11 | 0.63 (0.35-1.13) |  | 0.72 (0.47-1.10) | | |  | 1.33 (0.59-3.01) |  | 0.77 (0.47-1.26) |  |
| Vascular invasion |  | 0.194 |  | | | 0.010 |  | 0.108 |  | 0.002 |
| Negative | 1.00 |  | 1.00 | | |  | 1.00 |  | 1.00 |  |
| Positive | 2.34 (0.65-8.45) |  | 4.15 (1.41-12.26) | | |  | 6.96 (0.65-74.41) |  | 5.60 (1.86-16.82) |  |
| Neural invasion |  | 0.503 |  | | | 0.724 |  | 0.920 |  | 0.806 |
| Negative | 1.00 |  | 1.00 | | |  | 1.00 |  | 1.00 |  |
| Positive | 1.37 (0.54-3.46) |  | 1.15 (0.53-2.47) | | |  | 1.09 (0.21-5.66) |  | 0.89 (0.36-2.23) |  |
| CRM invasion |  | 0.749 |  | | | 0.702 |  | 0.098 |  | 0.944 |
| Negative | 1.00 |  | 1.00 | | |  | 1.00 |  | 1.00 |  |
| Positive | 1.45 (0.15-13.95) |  | 1.52 (0.18-13.01) | | |  | 8.58 (0.67-109.6) |  | < 0.001  (< 0.01, > 200) |  |

Note: ^#^Multivariate Cox regression model controlling for cT, cN, interval chemotherapy, surgical procedure, tumor grade, TRG score, ypT, ypN, No. of LN examined, vascular invasion, neural invasion and CRM.

Abbreviation: HR, hazard ratio; TD, tumor deposit; LN, lymph node; TRG, tumor regression grade; CRM, circumferential resection margin.

**Table S4.** Survival disparities in patients with different positive LN numbers stratified by TD subgroups

| Positive LN numbers | OS | DFS | LRFS | DMFS |
| --- | --- | --- | --- | --- |
| 1 | 0.010 | 0.148 | 0.013 | 0.622 |
| 2 | 0.716 | 0.810 | 0.517 | 0.662 |
| 3 | 0.348 | 0.555 | 0.086 | 0.744 |
| ≥ 4 | 0.671 | 0.668 | 0.749 | 0.451 |

Note: All P-values were calculated by Kaplan-Meier method.

Abbreviation: LN, lymph node; OS, overall survival; DFS, disease free survival; LRFS, local recurrence free survival; DMFS, distant metastasis free survival.

**Table S5.** Definition of TD grade

| TD grade | TD number | Patient number |
| --- | --- | --- |
| TD 0 | 0 | 407 |
| TD 1 | 1 | 39 |
| TD 2 | 2-3 | 29 |
| TD 3 | ≥ 4 | 20 |

Abbreviation: TD, tumor deposit.

**Table S6.** Associations between TD numbers and survival in all, LN-negative and LN-positive LARC patients.

| Patients | Survival | Kaplan-Meier  (P-value*) | Multivariate survival analyses^#^ | | | |
| --- | --- | --- | --- | --- | --- | --- |
|  |  |  | HR  (1 vs. 0) | HR  (2 vs. 0) | HR  (3 vs. 0) | P-value |
| All patients | OS | ＜0.001 | 1.64 | 1.52 | 2.34 | 0.062 |
|  | DFS | ＜0.001 | 1.14 | 0.79 | 0.82 | 0.804 |
|  | LRFS | 0.003 | 3.27 | 0.59 | 2.54 | 0.013 |
|  | DMFS | 0.002 | 1.48 | 1.46 | 1.33 | 0.414 |
| LN-negative  patients | OS | ＜0.001 | 1.56 | 3.40 | 3.56 | 0.026 |
|  | DFS | ＜0.001 | 1.26 | 3.31 | 5.88 | ＜0.001 |
|  | LRFS | 0.158 | - | - | - | - |
|  | DMFS | ＜0.001 | 1.15 | 4.06 | 3.10 | 0.003 |
| LN-positive  patients | OS | 0.857 | - | - | - | - |
|  | DFS | 0.660 | - | - | - | - |
|  | LRFS | 0.012 | 6.84 | 0.47 | 1.34 | 0.009 |
|  | DMFS | 0.709 | - | - | - | - |

Note: *P-values were calculated by Kaplan-Meier method. ^#^Multivariate Cox regression model controlling for cT, cN, interval chemotherapy, surgical procedure, tumor grade, TRG score, ypT, ypN (only in LN positive patients), No. of LN examined, vascular invasion, neural invasion and CRM.

Abbreviation: HR, hazard ratio; LN, lymph node; OS, overall survival; DFS, disease free survival; LRFS, local recurrence free survival; DMFS, distant metastasis free survival.

**Table S7.** Distribution of patients in N staging subgroups calculated by three N staging methods

| N subgroups | | oN method | | n1N method | | n2N method | |
| --- | --- | --- | --- | --- | --- | --- | --- |
| N0 | | 347 | | 307 | | 307 | |
| N1 | N1a | 100 | 44 | 140 | 44 | 118 | 52 |
|  | N1b |  | 56 |  | 56 |  | 66 |
|  | N1c |  | 0 |  | 40 |  | 0 |
| N2 | N2a | 48 | 33 | 48 | 33 | 70 | 46 |
|  | N2b |  | 15 |  | 15 |  | 24 |

**Table S8.** Survival disparities between LN-TDi+ and LNj+TD- (i = j) patients

| Groups | OS | DFS | LRFS | DMFS |
| --- | --- | --- | --- | --- |
| i = j = 1 | 0.864 | 0.850 | 0.637 | 0.613 |
| i = j = 2 | 0.234 | 0.481 | 0.200 | 0.373 |

Note: All P-values were calculated by Kaplan-Meier method. I, number of positive TDs; j, number of positive lymph nodes.

Abbreviation: OS, overall survival; DFS, disease free survival; LRFS, local recurrence free survival; DMFS, distant metastasis free survival.
